# Structure-Function Characterization of the Conserved Regulatory Mechanism of the Escherichia coli M48 Metalloprotease BepA

**DOI:** 10.1128/JB.00434-20

**Published:** 2020-12-18

**Authors:** Jack A. Bryant, Ian T. Cadby, Zhi-Soon Chong, Gabriela Boelter, Yanina R. Sevastsyanovich, Faye C. Morris, Adam F. Cunningham, George Kritikos, Richard W. Meek, Manuel Banzhaf, Shu-Sin Chng, Andrew L. Lovering, Ian R. Henderson

**Affiliations:** a Institute of Microbiology and Infection, University of Birmingham, Edgbaston, United Kingdom; b Department of Chemistry, National University of Singapore, Singapore, Singapore; c Institute for Molecular Bioscience, University of Queensland, St. Lucia, Australia; Princeton University

**Keywords:** *Escherichia coli*, structure, BepA, lipopolysaccharide, LptD, M48 metalloprotease, BAM complex, outer membrane, protease

## Abstract

The asymmetric Gram-negative outer membrane (OM) is the first line of defense for bacteria against environmental insults and attack by antimicrobials. The key component of the OM is lipopolysaccharide, which is transported to the surface by the essential lipopolysaccharide transport (Lpt) system. Correct folding of the Lpt system component LptD is regulated by a periplasmic metalloprotease, BepA. Here, we present the crystal structure of BepA from Escherichia coli, solved to a resolution of 2.18 Å, in which the M48 protease active site is occluded by an active-site plug. Informed by our structure, we demonstrate that free movement of the active-site plug is essential for BepA function, suggesting that the protein is autoregulated by the active-site plug, which is conserved throughout the M48 metalloprotease family. Targeted mutagenesis of conserved residues reveals that the negative pocket and the tetratricopeptide repeat (TPR) cavity are required for function and degradation of the BAM complex component BamA under conditions of stress. Last, we show that loss of BepA causes disruption of OM lipid asymmetry, leading to surface exposed phospholipid.

**IMPORTANCE** M48 metalloproteases are widely distributed in all domains of life. E. coli possesses four members of this family located in multiple cellular compartments. The functions of these proteases are not well understood. Recent investigations revealed that one family member, BepA, has an important role in the maturation of a central component of the lipopolysaccharide (LPS) biogenesis machinery. Here, we present the structure of BepA and the results of a structure-guided mutagenesis strategy, which reveal the key residues required for activity that inform how all M48 metalloproteases function.

## Abstract (precis)

M48 metalloproteases are widely distributed in all domains of life. E. coli possesses four members of this family located in multiple cellular compartments. The functions of these proteases are not well understood. Recent investigations revealed that one family member, BepA, has an important role in the maturation of a central component of the lipopolysaccharide (LPS) biogenesis machinery. Here, we present the structure of BepA and the results of a structure-guided mutagenesis strategy, which reveal the key residues required for activity that inform how all M48 metalloproteases function.

## INTRODUCTION

The outer membrane (OM) of Gram-negative bacteria is the first line of defense against environmental insults, such as antimicrobial compounds (1, 2). As such, the integrity of the OM must be maintained lest the bacteria become susceptible to stresses to which they would otherwise be resistant. The OM consists of an asymmetric bilayer of phospholipids and lipopolysaccharide (LPS) decorated with integral outer membrane proteins (OMPs) and peripheral lipoproteins. The impermeable nature of the OM can be attributed to several characteristics of the LPS leaflet, such as dense acyl chain packing, intermolecular bridging interactions, and the presence of O-antigen carbohydrate chains (1, 3–7).

All the components required to construct the OM are synthesized in the cytoplasm. Specialized systems transport these molecules across the cell envelope and assemble the molecules into the OM in a coordinated fashion. Central to this is the β-barrel assembly machinery (BAM) complex. In Escherichia coli, the BAM complex is composed of two essential subunits, the OM β-barrel BamA and the lipoprotein BamD, and three nonessential accessory lipoproteins, BamB, BamC, and BamE (8–11). The BAM complex is responsible for assembly of the Lpt system, which traffics LPS from the cytoplasm to the outer leaflet of the OM in order to maintain OM permeability barrier function (12–14). The Lpt machinery is composed of three modules: the inner membrane (IM)-localized LptBFGC complex, which flips the LPS molecule across the IM and energizes the system; LptA, which forms a bridge between the IM and OM along which the LPS travels; and the OM complex LptDE (12, 15, 16). The C terminus of LptD forms an OM β-barrel which facilitates insertion of LPS directly into the outer leaflet of the OM (17, 18). The N terminus contains a periplasmic domain that interacts with the LptA bridging molecule (19). The two LptD domains are connected via two disulfide bonds, at least one of which is required for efficient function of the LptDE complex (20, 21). Formation of the correct LptD disulfide bonds is reliant upon the periplasmic thiol-disulfide oxidoreductase, DsbA, as well as proper folding and insertion of the LptD β-barrel into the OM. The latter step is dependent on the BAM complex and the interaction of LptD with its cognate OM lipoprotein partner LptE (20, 21). To be effective at LPS delivery and to maintain the integrity of the OM, maturation of the LptDE complex is tightly regulated. The proteases DegP, BepA, and YcaL each have specific roles in LptD maturation. DegP is responsible for the degradation of misfolded LptD in the periplasm, whereas YcaL targets LptD which has docked with the Bam machinery but stalled at an early step in folding. Lastly, BepA degrades LptD which has engaged with the Bam machinery but stalled during insertion of a nearly complete barrel (22).

Among the LptD quality control proteases, BepA is different in that it also has chaperone activities and influences the insertion of other OMPs into the outer membrane, and its deletion renders cells sensitive to multiple antibiotics (23, 24). The primary sequence of BepA indicates that this protein is a member of the M48 family of zinc metalloproteases, of which there are four in E. coli. The M48 proteases are characterized by an HEXXH motif on the active-site helix (25). The histidine residues within this motif act, usually with a third amino acid and a water molecule, to coordinate the metal ion, typically zinc, at the active site (26–28). In addition to the N-terminal M48 protease domain, BepA has a C-terminal tetratricopeptide repeat (TPR) domain. TPR domains consist of a number of stacked repeats of α-helix pairs, together forming a solenoid-like structure that is known to facilitate protein-protein interactions and multiprotein complex formation (29). Narita et al. reported that BepA has a dual role, degrading misfolded LptD but also promoting correct folding and accumulation of the mature disulfide isomer of LptD (23). Further to this, the BepA protease has been shown to interact with the main BAM complex component, BamA, and to degrade BamA under conditions of stress created by the absence of the periplasmic OMP chaperone SurA (23).

Following the work of Narita et al. (23), we sought to determine the structure of BepA to understand the roles of the TPR and M48 peptidase domains in substrate recognition and processing. During this study, two papers from other groups were published using similar structural approaches. First, Daimon et al. (30) determined the crystal structure of the TPR domain of BepA in isolation and observed that this domain presents a negatively charged face which was postulated to recognize components of the Bam complex and LptD. Using protein cross-linking analysis, they demonstrated that residues of the TPR domain interacted with BamA, BamC, BamD, and LptD. Mutation of the TPR residue F404 resulted in decreased proteolysis of BamA, indicating that this residue is involved in targeting of the M48 protease domain of BepA to this substrate. More recently, Shahrizal et al. (31) presented a full-length structure of the BepA TPR and M48 protease domains solved to 2.6 Å. In this structure, the negatively charged TPR face noted by Daimon et al. (30) is largely buried, forming a peripheral association with the M48 protease domain. SAXS and engineered disulfide bonds were used to explore the potential for the TPR domain and M48 domain to move relative to one another, but the cross-linking experiments demonstrated that the TPR and M48 domains likely remain in tight association. While multiple mutations were made, designed from the full-length structure of BepA, none of them led to any significant observable phenotype when expressed in E. coli (30).

Here, we present our independently solved 2.19-Å structure of near-full-length BepA, encompassing the TPR and M48 domains. Our structure largely agrees with that of Shahrizal et al. (31), providing further evidence that TPR movement relative to the M48 domain is unlikely to be a mechanism of BepA function. Additionally, we noted the presence of an active-site plug, the TPR cavity, and the negatively charged pocket formed by the association of the BepA TPR and M48 domains, which we targeted for further study. Using structure-led mutagenesis studies, we probed the roles of these three BepA structural elements and identified key residues in each that are required for BepA function. Furthermore, the active-site plug of BepA is a structural element conserved in the M48 protease family, and so our findings have broad ramifications for proteases involved in processing varied substrates across all domains of life.

## RESULTS

### The BepA structure reveals a nautilus-like structure with TPR-protease contacts.

**FIG 1:**
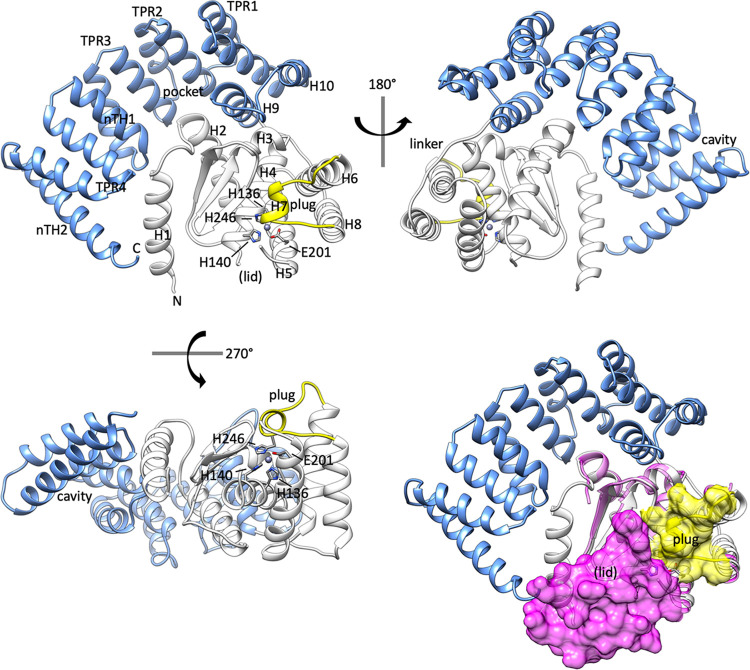
The structure of BepA reveals an occluded active site. Cartoon schematic of the X-ray crystallography structure of BepA, solved to a resolution of 2.18 Å. The TPR domain is represented in blue and the protease domain in white, with the active-site plug in yellow. Also labeled are the N and C termini, the TPR pocket, the TPR cavity, the linker and the site at which we expect the active site lid (lid). Important active site residues H136, H140, H246, and E201 are shown by a stick diagram. TPR motifs 1 to 4, non-TPR helices 1 and 2 (nTH1 and nTH2), helices, sheets, and the plug are labeled. Alignment of the structure presented here with that of the G. sulfurreducens M48 metalloprotease (PDB code 3C37; magenta ribbon) reveals occlusion of the active site by the potential active site lid, represented as magenta surface density from the 3C37 structure; the active-site plug is represented as yellow surface density.

The crystal structure of BepA_L44–Y484_ was solved to a resolution of 2.18 Å by experimental phasing using the endogenous zinc copurified with recombinant BepA protein, present in our structure at a 1:1 stoichiometry with BepA (data collection and refinement statistics are reported in Table 1); we observe a single copy of BepA in the asymmetric unit. The structure revealed the TPR domain, consisting of 12 α-helices forming 4 TPR motifs and four non-TPR helices, in tight association with the M48 zinc-metallopeptidase domain. This forms a nautilus-like fold with the TPR subdomain cupping the metallopeptidase domain (Fig. 1). The high-resolution data presented here are in broad agreement with those presented previously (30, 31); however, there are some differences of note. The BepA TPR subdomain was previously annotated as being composed of four TPR motif helix pairs and two non-TPR helices (nTH1 and nTH2); therefore, we have adopted this nomenclature.

**TABLE 1:** Data collection and refinement statistics

Data category	Value(s)^*a*^
Data collection	
Space group	P21 21 21
Cell dimensions	
*a*, *b*, *c* (Å)	53.12, 77.02, 124.60
α, β, γ (°)	90.00, 90.00, 90.00
Resolution (Å)	77.02–2.18
*I*/σ*I*	2.65 (at 2.18 Å)
Completeness (%)	98.9 (91.5)
Redundancy	16.5 (6.0)

Refinement	
Resolution (Å)	2.18
No. of reflections	25,763
*R*_work_/*R*_free_	0.176/0.201
No. of atoms	3,269
Protein	3,143
Ligand/ion	6
Water	120
*B* factors	47.0
Protein	43
Ligand/ion	71 (SO_4_), 37 (Zn)
Water	48.9
RMSD^*b*^	
Bond lengths (Å)	0.006
Bond angles (°)	1.00

a Values in parentheses are for highest-resolution shell.

b Root mean square deviation.

Our structure demonstrates that the TPR domain consists of 12 α-helices, whereas the structure was previously annotated with 10 α-helices in order to maintain the nomenclature used with the previously solved TPR domain structure of residues 310 to 482 (30, 31). Despite their previous annotation as non-TPR helices, we observe that helices 8 and 9 form part of the TPR domain and are preceded by an extended linker region, comprising residues M263 to S271, which connects the TPR domain to helix 7 of the protease domain (Fig. 1). Helices 8 and 9 contribute a tight turn at the end of the TPR domain, allowing the M48 metallopeptidase domain to be cupped by the pocket formed from TPR motifs 2 and 3 (Fig. 1). Interaction of the protease domain with the TPR pocket creates a larger negatively charged pocket, which is also noted in the structure presented by Shahrizal et al. (31). The context provided by the full-length protein structure shows that while the TPR pocket interacts with the protease domain, the TPR cavity is positioned away from the protease active site on the opposite side (Fig. 1). The cavity also comes into close proximity to the N-terminal helix, which is contained within the protease domain, therefore potentially facilitating TPR-protease domain communication.

The protease domain of BepA consists of the active site α-helix H4 containing the HEXXH motif, and an active-site plug formed by a loop between helices H6 and H7, residues S246 to P249. We did not observe any density corresponding to positions L146 to I194, and considering that this section is in close proximity to the active site, we expect that it may form a dynamic regulatory region (Fig. 1). We sought evidence that the unresolved area of the protein may correspond to a dynamic lid. Therefore, we scrutinized the Protein Data Bank (PDB) for similar structures. Information on the missing region of our structure can be inferred from an unpublished structure in the PDB of an M48 zinc-metallopeptidase from Geobacter sulfurreducens, which consists of only the protease domain, with no associated TPR (PDB code 3C37). The structure of the G. sulfurreducens protease structure provides some information on the missing section and demonstrates a short three-turn extension to the C terminus of active-site helix H4, beyond that seen in the BepA structure. This is followed by a glycine-facilitated kink and another three helical turns terminating at residue D136 of the 3C37 structure (see Fig. S1 in the supplemental material). The 3C37 structure is also missing a section, D136 to N139; however, residues M140 to F149 form another short α-helical region, which is connected to the N terminus of helix H5 by an extended region formed by residues G150 to S158 of the 3C37 structure (Fig. S1). While also incomplete, the recently published BepA structure also provides some information on this section, which is also largely in agreement with that of the 3C37 structure (Fig. S1) (31). Overall, comparison of the structure presented here, that of Shahrizal et al. (31) (PDB code 6AIT), and the G. sulfurreducens structure (PDB code 3C37) suggests that the missing section from the structure presented here may form a putative active-site lid. The putative lid, along with the plug, likely regulates access to the active site, as alignment of the three structures shows that the lid and plug occlude the active site (Fig. 1). The facts that no density for the putative lid is observed in our structure and that partial sections are missing in those presented previously suggest that the active-site lid is dynamic and may adopt multiple conformations.

### Mobility of the conserved active-site plug is required for BepA function.

**FIG 2:**
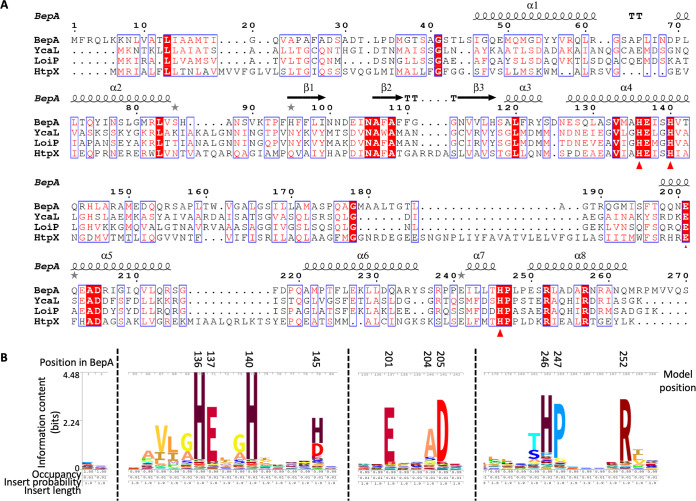
Conservation of the M48 metalloprotease HEXXH motif and active-site plug residues. (A) Amino acid sequences for E. coli BepA, YcaL, LoiP, and HtpX were submitted to Clustal Omega (https://www.ebi.ac.uk/Tools/msa/clustalo/) in order to generate a multiple alignment to allow analysis of amino acid conservation and subsequently processed using ESPript 3.0 (http://espript.ibcp.fr) (53, 54). Sequence numbering (above the line) is based on the BepA sequence. Gaps in the alignment are represented by dots. Single fully conserved residues are highlighted in red, and the zinc coordinating residues are labeled with a triangle under the residue. BepA secondary structure is represented on the top line with α-helices indicated by spirals and β-sheets indicated by arrows. The protease domain subsection of the alignment is shown (for the full alignment, see Fig. S3). (B) HMM logo generated for the pfam M48 family of metalloproteases (PF01435) from the pfam website (https://pfam.xfam.org) with the HMM profile constructed on the pfam seed alignment. Three sections of the alignment are shown, which demonstrate conservation of the active site zinc coordinating residues H136, H140, E201, and H246. The active-site plug clearly contains a conserved motif, HPX_4_R. Amino acid position in BepA and within the model are both shown, with the stack height corresponding to information content (bits), which represents the invariance of the position. Letter height divides the stack height according to letter frequency.

**FIG 3:**
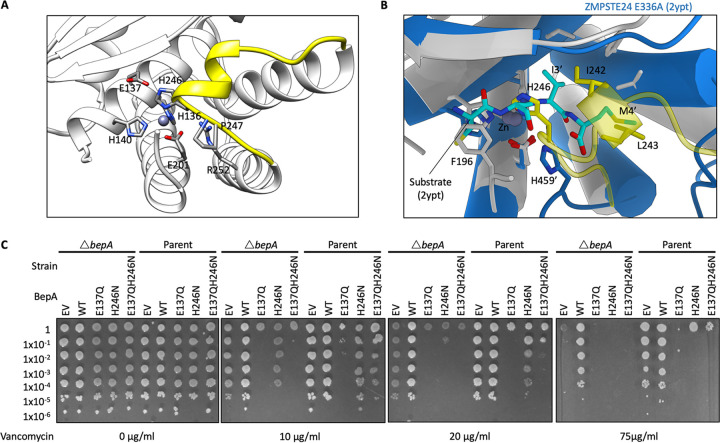
The BepA active-site plug acts to regulate BepA proteolytic activity. Analysis of the BepA structure suggested a regulatory role for the active-site plug; therefore, plasmids carrying mutated *bepA* were screened for their capacity to complement the vancomycin sensitivity of Δ*bepA* E. coli. (A) Structural diagram of the BepA active site with key conserved residues represented by a stick diagram and labeling. (B) Alignment of the BepA active site (transparent white and yellow ribbon) with that of the human nuclear membrane zinc metalloprotease ZMPSTE24 mutant E336A with a synthetic substrate peptide (PDB code 2YPT) (55) (opaque blue ribbon). 2YPT residues are labeled with the addition of a prime symbol. (C) Screen for vancomycin sensitivity of cells carrying pET20b encoding WT or mutated copies of BepA in the parent or Δ*bepA* strain background. EV, empty vector control. Cells were normalized to an OD_600_ of 1 and 10-fold serially diluted before being spotted on the LB agar containing the indicated antibiotics (all plates also contained 100 μg/ml carbenicillin).

The structure shows the HEXXH motif, which is characteristic of zinc-dependent metallopeptidases (25, 27) and is found within helix H4 (Fig. 1). The active-site zinc ion is coordinated by H136 and H140 within the HEXXH motif, E201 contributed by helix H5, and H246 on a loop that forms the small α-helical active-site plug (Fig. 1). Multiple alignment of the four E. coli M48 metallopeptidases, HtpX, YcaL, LoiP, and BepA, demonstrates that zinc-coordinating residues are all conserved, along with the proline following H246, P247, and an arginine further toward the C terminus, R252, which resides within the active site (Fig. 2A and Fig. 3A). Analysis of the hidden Markov model (HMM) logo generated for the M48 metallopeptidase family demonstrated that not only are the HEXXH motif and the zinc-coordinating glutamic acid conserved, but the HPX_4_R motif within the active-site plug is also conserved throughout the whole pfam family (PF01435), which includes proteins from all domains of life (Fig. 2B). Addition of the active-site plug motif to the characteristic HEXXH motif of zinc metallopeptidases allows the specific identification of this protein family within E. coli by using the online pattern search tool MOTIF2 (https://www.genome.jp/tools/motif/MOTIF2.html) using the pattern search H-E-x-x-H-x(30, 140)-H-P-x(4)-R. The results of this search, in E. coli, identify three proteins other than the four M48 family metallopeptidases. One of these is a prophage cell death peptidase encoded by the *lit* gene, which is classified as the single member of the U49 peptidase family. We anticipate that this peptidase and the rest of the M48 family of zinc metallopeptidases are likely to contain the HP motif active-site plug structural element, similar to that of BepA.

The active-site zinc ion is usually chelated by three amino acid residues and one water molecule, which is utilized to catalyze proteolysis of the substrate (26, 28). Coordination of the zinc ion by H246 acts as the fourth ligand, therefore suggesting that a rearrangement of the active-site plug should be required for proteolytic activity. Alignment of the structure of human nuclear membrane zinc metalloprotease, ZMPSTE24, with a bound substrate peptide (PDB code 2YPT) reveals that the BepA active-site plug occupies the physical space that the substrate for ZMPSTE24 would occupy (Fig. 3B). Residue H246 on the BepA active-site plug directly clashes with positioning of substrate in the 2YPT structure, and the hydrophobic residues I242 and L243 occupy a space similar to that of the 2YPT substrate hydrophobic residues I3′ and M4′ (Fig. 3B). Based on these observations, we hypothesized that the active-site plug occludes the active site and is likely to relocate in order to facilitate substrate access to the active site (Fig. 1 and Fig. 3B).

To test the importance of H246 in occupying the fourth coordination site on the zinc ion, we generated a mutation of the H246 position to asparagine (H246N). For comparison, we also constructed the E137Q mutation in the active helix HEXXH motif, which has previously been shown to prevent protease activity of BepA (23). To test whether the H246N BepA mutant is functional, we assayed the ability of this mutant to complement the Δ*bepA* strain, which is known to exhibit increased sensitivity to large antibiotics such as vancomycin, presumably due to impaired barrier function of the OM. The E137Q active-site mutant was incapable of restoring vancomycin resistance to Δ*bepA* cells and severely increased the vancomycin sensitivity of the Δ*bepA* mutant (Fig. 3C). The H246N mutant BepA was also incapable of complementing vancomycin sensitivity of the Δ*bepA* cells; however, while the H246N protein also severely increased the vancomycin sensitivity of the mutant beyond that of the empty vector control, the negative effect was less extreme than with the E137Q version of the protein (Fig. 3C). Considering this phenotype, we decided to investigate if the mutated proteins had a dominant negative effect in the parent background expressing wild-type *bepA*. We found that the empty vector and wild-type BepA had no detrimental effect on BW25113 parent cells grown in the presence of vancomycin. Our analysis of the E137Q mutant was in agreement with previous studies, where it was analyzed in the parent background and demonstrated a severe dominant negative phenotype (23). We also observed that the presence of H246N BepA had a dominant negative effect on the capacity of the cells to grow in the presence of vancomycin, despite the presence of wild-type BepA expressed from the chromosomal locus. Similar to the effect in the mutant background, the dominant negative effect of the H246N protein was less severe than that of the E137Q derivative (Fig. 3C). Western blotting to detect the expression of BepA proteins in whole-cell lysates using anti-His_6_ antibodies showed an elevated level of the E137Q protein compared to the wild type and an absence of observable tagged protein in the H246N sample. These observations were consistent between the Δ*bepA* and parent backgrounds (Fig. S2). These results support the hypothesis that the E137Q mutation renders BepA protease inactive, therefore stabilizing the protein due to a lack of autoproteolytic activity, which has been observed previously (23). Considering that the H246N BepA has a dominant negative effect, the absence of a detectable tagged protein by Western blotting suggests the C-terminal His tag may be autoproteolytically degraded, an observation that has previously been made for the wild-type BepA protein (23). These data support the hypothesis that the H246N mutation gives rise to a protein with deregulated proteolytic activity.

In order to test if the autoproteolysis of the H246N protein was due to increased protease activity, we introduced the established protease-dead mutation E137Q. The BepA E137Q H246N substitution was not capable of complementing the vancomycin sensitivity and had a severe dominant negative effect similar to that of E137Q alone (Fig. 3C). Analysis of the E137Q H246N BepA protein by Western blotting showed a similar level of tagged protein to the E137Q protein (Fig. S2). These data suggest that introduction of the E137Q mutation either prevents autoproteolysis of the C-terminal His_6_ tag in the H246N mutant or alternatively stabilizes the protein, preventing it from being targeted by other periplasmic proteases.

**FIG 4:**
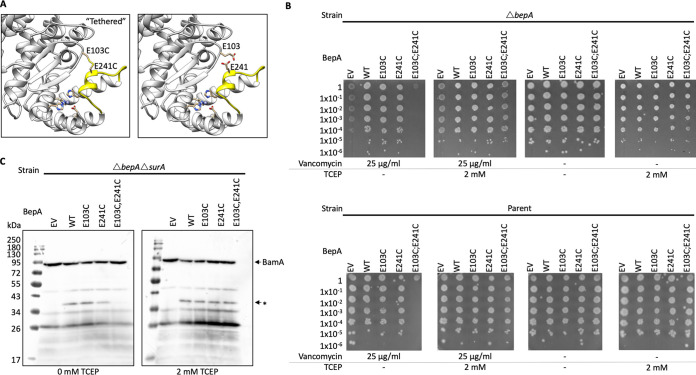
Flexibility of the active-site plug is required for BepA proteolytic activity. The requirement for flexibility of the BepA active-site plug for full BepA function was assayed by disulfide bond tethering of the active-site plug and functional screening. (A) Structural representation of the BepA active site with residues targeted for mutation to cysteine, E103 and E241, labeled and colored yellow. (B) Screen for vancomycin sensitivity of cells carrying pET20b encoding WT or mutated copies of BepA in the parent or Δ*bepA* strain background. EV, empty vector control. Cells were normalized to an OD_600_ of 1 and 10-fold serially diluted before being spotted on the LB agar containing the indicated antibiotics or the reducing agent TCEP (all plates also contained 100 μg/ml carbenicillin). (C) The E103C E241C double cysteine substitution prevents BepA-dependent generation of a putative BamA degradation product in the Δ*surA* background in the absence of TCEP. Total cellular protein extracts were prepared from Δ*bepA* Δ*surA* cells carrying pET20b encoding WT or mutated copies of BepA following growth in the presence or absence of TCEP. EV, empty vector control. Samples were separated by SDS-PAGE and transferred to nitrocellulose membrane for Western immunoblotting using antisera raised in rabbits against the BamA POTRA domain. The putative BamA degradation product is labeled with an asterisk, and the full-length BamA is indicated.

The importance of residue H246 for BepA function, and the conformation of the active-site plug observed in our crystal structure, suggests that the solved crystal structure may represent a proteolytically inactive form of the protein. Therefore, we hypothesized that movement of the active-site plug must be required to facilitate substrate access to the active site. We aimed to tether the active site in the conformation observed in our crystal structure by engineering a disulfide bond. Cysteine substitutions were introduced into proximal sites in BepA, specifically at positions E103, in the loop between S1 and S2, and E241 in the active-site plug, either individually or in concert (Fig. 4A). The single-cysteine-substitution mutants complemented the vancomycin sensitivity phenotype, indicating that the single substitutions had no impact on BepA function. However, the double cysteine mutant was incapable of restoring vancomycin resistance to the *bepA* mutant under normal growth conditions. In contrast, in the presence of the reducing agent TCEP [tris(2-carboxyethyl)phosphine], the double cysteine mutant was able to complement vancomycin sensitivity (Fig. 4B). The double cysteine mutant also caused a severe dominant negative effect in the parent background, which was alleviated by the presence of the reducing agent TCEP (Fig. 4B). These observations suggest that in the E103C E241C BepA, a disulfide bond may have formed that tethered the active-site plug in an inactive conformation, causing effects similar to those of the E137Q protease-dead mutation, and that free movement of the plug is essential to function (Fig. 4B).

BepA has been shown to degrade the BAM complex component BamA under conditions of stress induced by the absence of the chaperone SurA (23). Therefore, we analyzed whole-cell lysates from Δ*bepA* Δ*surA* cells expressing wild-type (WT) BepA or the E103C, E241C, or E103C E241C double mutant derivatives of BepA by Western immunoblotting with antiserum raised against POTRA (polypeptide transport-associated) domain 5 of BamA (32). Cells were grown in the presence or absence of the reducing agent TCEP. As demonstrated previously, we observed that introduction of WT BepA into the cells leads to generation of an anti-BamA antibody-reactive BamA degradation product of approximately 40 kDa, regardless of TCEP addition to the growth medium (23) (Fig. 4C). Production of the putative BamA degradation product was also detected in cells expressing the single cysteine substitutions. However, BepA-dependent BamA degradation was decreased in cells expressing the double cysteine mutant under standard growth conditions but was restored during growth in the presence of TCEP (Fig. 4C). These results demonstrate that *in vivo* BepA proteolytic degradation of the substrate BamA requires free movement of the active-site plug.

### The BepA-negative pocket and TPR cavity are required for function and BepA-mediated degradation of BamA.

**FIG 5:**
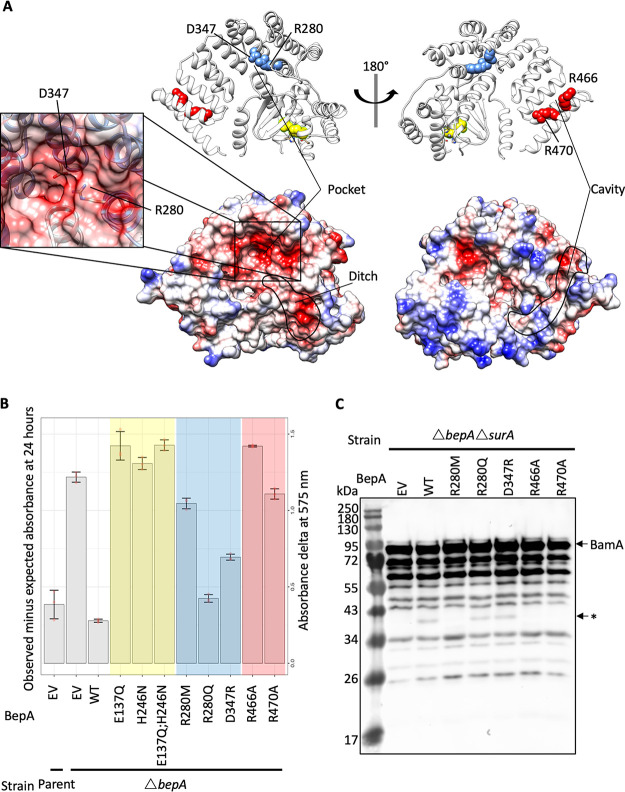
Conserved residues in the pocket and TPR cavity are required for function. The BepA pocket and TPR cavity contain conserved residues that were mutated to test their importance for BepA function. (A) Structure of BepA showing residues targeted for mutation as color-coded spheres that match the color-coded chart in panel B. Also shown are surface representations of BepA in the same orientations, colored according to surface charge: red for negatively charged, white for near neutral, and blue for positive charge. The enlargement in the box depicts the position of the key D347 and R280 residues. (B) CPRG permeability assay of parent or Δ*bepA* cells carrying pET20b with WT or mutant copies of BepA, as indicated. EV, empty vector control. The CPRG turnover score (change in absorbance at OD_575_ compared to Lac^−^ cells) is represented for two independent experiments each containing three replicates. (C) The R280M, R466A, and R470A substitutions prevent BepA-dependent generation of a putative BamA degradation product in the Δ*surA* background. Total cellular protein extracts were prepared from Δ*bepA* Δ*surA* cells carrying pET20b encoding WT or mutated copies of BepA. EV, empty vector control. Samples were separated by SDS-PAGE and transferred to nitrocellulose membrane for Western immunoblotting using antisera raised in rabbits against the BamA POTRA domain. The putative BamA degradation product is labeled with an asterisk, with the full-length BamA indicated.

The TPR domain contains two potential substrate binding sites, termed the “pocket” on the protease-proximal face and the “cavity” on the protease-distal face (Fig. 1 and 5). We identified two conserved charged residues, R280 and D347, in the BepA TPR pocket, which forms a larger negatively charged cleft through interaction with the protease domain (Fig. 5A). The negatively charged cleft is connected to the active site via a negatively charged ditch and has previously been hypothesized to facilitate substrate interactions (31). However, no evidence for the importance of this site for BepA function has yet been provided. We targeted these two conserved charged residues within the pocket, and vancomycin sensitivity assays revealed that the R280 mutations had no significant effect on complementation of the *bepA* mutant. However, the D347R mutation had a mild negative effect on the capacity of the BepA protein to complement the vancomycin sensitivity of the *bepA* mutant and a dominant negative effect in the parent background. The D347R mutation led to the protein being detected at a lower level by immunoblotting than the WT protein, which could be due to either decreased stability or increased autoproteolytic activity (Fig. S2 and S3).

The effect of D347R is weak by comparison with that of the active-site mutations; therefore, we utilized a more sensitive permeability assay to assess the mutation. Vancomycin is a large (1,450-Da) hydrophobic antibiotic that does not normally penetrate the OM. The target for vancomycin is the abundant d-alanyl-d-alanine substrate, which it must bind in sufficient quantity to exhibit an effect on cell growth and lysis. Chlorophenyl red-β-d-galactopyranoside (CPRG) is a hydrophobic β-galactosidase substrate that is smaller (585 Da) but also fails to penetrate wild-type E. coli. OM permeability defects allow penetration of CPRG into the cell, where it is then accessible to cytoplasmic β-galactosidase, which hydrolyzes the CPRG to produce a red color (33, 34). Production of the red color is a sensitive indicator of cell permeability and thus can be measured using a time-resolved wave scan of cells grown on LB agar supplemented with CPRG (34, 35). The BW25113 parent strain is Lac^−^; therefore, strains were cotransformed with the relevant *bepA*-carrying plasmid and a *lacZYA* expression vector (33, 34, 36). CPRG assays indicated that the *bepA* mutant cells are indeed more permeable to the β-galactosidase substrate CPRG and that this permeability phenotype can be complemented (Fig. 5B). The active-site mutations E137Q and H246N, which cause increased vancomycin sensitivity compared to the *bepA* mutant empty vector control, are not able to restore the OM barrier against CPRG. The conditions used for the assay here appear to be too sensitive to measure the differences between the empty vector control and the E137Q and H246N mutants that are apparent from vancomycin sensitivity screening (Fig. 5B). However, the increased sensitivity of the assay showed that mutations altering conserved residues in the pocket (R280M and D347R) are not able to fully complement the permeability defect (Fig. 5B). This suggests that the phenotypes caused by these mutations are mild compared to those caused by the active-site mutations. The mild permeability phenotype could explain the lack of observable vancomycin sensitivity despite increased permeability to CPRG.

We next sought to assess the cavity in the TPR domain, which has been shown to be involved in BepA binding to the Bam complex (30). Conservation analysis revealed two conserved arginine residues, R466 and R470, which have yet to be analyzed for their role in BepA function. We expected that these residues might be involved in substrate recognition or interaction with protein complex partners due to the prominent position in the cavity and their high level of conservation despite the lack of any obvious structural role (Fig. 5A). Mutation of these residues to alanine appeared to have no impact on the capacity of the BepA protein to complement the vancomycin sensitivity phenotype (Fig. S3). However, the CPRG permeability assay demonstrated that R466 and R470 are indeed required for full complementation of the OM permeability defect caused by loss of BepA (Fig. 5B).

Considering that the TPR cavity has been shown to interact with Bam complex subunits (30) and that we observed cell permeability defects on complementation with the TPR cavity mutants, we reasoned that these mutants may be defective in BepA-mediated degradation of BamA. Therefore, we analyzed whole-cell lysates from Δ*bepA* Δ*surA* cells expressing WT BepA or the R280M, R280Q, D347R, R466A, or R470A derivatives of BepA by Western immunoblotting with anti-BamA antiserum (32). Production of the putative BamA degradation product was not detectable in cells expressing the R280M derivative or in cells expressing BepA with substitutions in the TPR cavity (R466A and R470A), all of which had the most severe permeability defects in this set (Fig. 5B and C). In contrast, production of the putative BamA degradation product was unaffected in cells expressing the negative pocket derivatives R280Q and D347R, which were also less permeable to CPRG than the other mutants assayed (Fig. 5B and C). These data suggest that these residues are important for BepA-mediated degradation of BamA in the absence of the chaperone SurA. This also supports the previous observation that the TPR cavity is required for interaction with the Bam complex (30).

### Loss of BepA leads to increased surface-exposed phospholipid.

**FIG 6:**
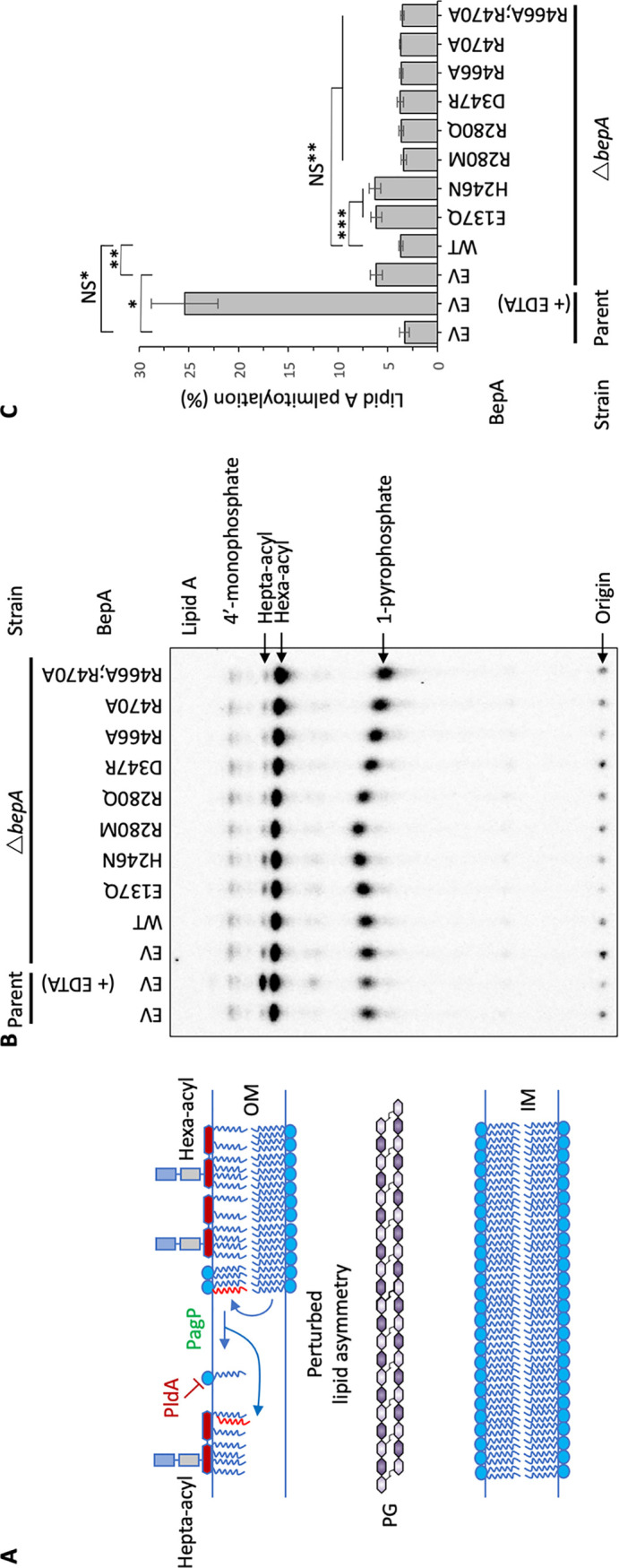
Loss of BepA leads to surface-exposed phospholipid. The increased permeability of Δ*bepA* cells was hypothesized to be due to increased surface-exposed phospholipid; therefore, this was tested by the PagP mediated lipid A palmitoylation assay, which detects surface exposed phospholipid. (A) Schematic demonstrating the role of PagP in sensing and responding to surface exposed phospholipid. (B) PagP-mediated lipid A palmitoylation assay. PagP transfers an acyl chain from surface-exposed phospholipid to hexa-acylated lipid A to form hepta-acylated lipid A. ^32^P-labeled lipid A was purified from cells grown to mid-exponential phase in LB broth with aeration. Equal amounts of radioactive material (counts per minute per lane) were loaded on each spot and separated by thin-layer chromatography before quantification. As a positive control, cells were exposed to 25 mM EDTA for 10 min prior to lipid A extraction in order to chelate Mg^2+^ ions and destabilize the LPS layer, leading to high levels of lipid A palmitoylation. (C) Hepta-acylated and hexa-acylated lipid A were quantified, and hepta-acylated lipid A levels are presented as a percentage of the total. Triplicate experiments were utilized to calculate averages and standard deviations, with Student’s *t* test used to assess significance. *, *P < *0.005 compared with parent empty vector control (EV); **, *P < *0.005 compared with Δ*bepA* EV; ***, *P* < 0.001 compared with Δ*bepA* WT; NS*, *P* > 0.1 compared with parent EV (not significant); NS**, *P* > 0.1 compared with Δ*bepA* WT (not significant).

The permeability of Δ*bepA* cells was demonstrated previously by sensitivity to erythromycin and here with vancomycin, as well as by CPRG assay (23). This has been suggested to be due to perturbed OM lipid asymmetry, which can be detected through monitoring the activity of the OM protein PagP, an enzyme that palmitoylates lipid A in response to surface-exposed phospholipid (37–39) (Fig. 6A). To measure the levels of hepta-acylated lipid A, radiolabeled lipid A was isolated from the Δ*bepA* mutant or bacteria that had been complemented with BepA, BepA E137Q, or BepA H246N. The lipids were then separated by thin-layer chromatography. The parent strain BW25113, transformed with empty pET20b, was treated with EDTA prior to lipid A isolation, a process that is known to induce high levels of hepta-acylated lipid A production and act as a positive control (40–42). Cells lacking BepA showed a significant increase in the levels of hepta-acylated lipid A in relation to hexa-acylated lipid A, indicating perturbation of OM lipid asymmetry in the absence of functional BepA (Fig. 6B and C). While the E137Q and H246N mutants were not able to rescue this defect, they also did not appear to significantly increase the levels of hepta-acylated lipid A compared to cells lacking BepA (Fig. 6B and C). Additionally, we did not see any effect on lipid A palmitoylation for any of the other mutations used in this study. This is likely due to the lack of sensitivity of the assay. These data demonstrate that loss of BepA leads to an increase in surface exposed phospholipid and subsequent modification of lipid A.

## DISCUSSION

In this study, we present the structure of full-length BepA at a resolution of 2.18 Å, which is a periplasmic M48 zinc metalloprotease family protein involved in regulating the maturation of the LPS biogenesis machinery in Gram-negative bacteria. It has been established that cells lacking BepA are more sensitive to hydrophobic antibiotics with a high molecular mass, such as vancomycin, erythromycin, rifampin, and novobiocin, which has been suggested to be caused by increased OM permeability (23). Here, we supply further evidence, obtained through the use of the CPRG assay, that the *bepA* mutant has increased cell permeability and show that there is an increased amount of surface-exposed phospholipid, as determined with the PagP reporter assay. Therefore, we hypothesize that the loss of BepA results in decreased LptD assembly, leading to decreased OM LPS content. This would in turn cause phospholipids to flip from the inner leaflet to the outer leaflet of the OM, creating a perturbation in OM lipid asymmetry and increased OM permeability to large antibiotics and the CPRG molecule (22, 23, 43). On detecting surface exposed phospholipids, the OM-localized lipid A palmitoyltransferase, PagP, utilizes the outer leaflet phospholipids as palmitate donors to convert hexa-acylated lipid A to hepta-acylated lipid A (37–39), as detected in this study. While this hypothesis is well supported by the data, we should also consider the possibility that increased OM permeability could be caused by defective, partially folded OMP barrels.

The solved crystal structures, presented here and previously, are missing density for a region near the active site, which we suggest is an active site lid that in part occludes access to the active site residues (31). The three available structures all demonstrate some missing density within the lid; this could be explained by flexibility within this region to facilitate substrate access to the active site. This highlights an attractive area for future study of the regulatory mechanisms employed by BepA and the M48 metalloproteases. In combination with the active site lid, access to the site is also blocked by the active-site plug, which we focused on here. We have shown that H246 within a small helix on the active site loop coordinates the zinc in our structure and is essential for correct function of BepA. We speculate that this may be because the active-site plug in the H246N mutant is less able to interact with the active site zinc ion and that the protein may be in a constitutively activated or “deregulated” conformation. The H246N derivative of BepA was undetectable through immunoblotting with antibodies targeting the C-terminal His tag, which would be positioned in close proximity to the active site in the solved crystal structure. This could be due to either increased C-terminal autoproteolysis of the His tag or increased instability of BepA H246N and subsequent degradation by other periplasmic proteases. Stabilization of the H246N mutant by introduction of the E137Q mutation supports the hypothesis that this mutant is hyperactivated; however, it does not rule out the possibility that the vancomycin sensitivity phenotype could be due to increased periplasmic degradation of BepA by other proteases. Regardless, our data demonstrate that the interaction of the plug with the active site zinc ion is essential for correct function of BepA. Through the use of disulfide bond tethering, we also demonstrate that the plug must be mobile for proteolytic activity of BepA on the *in vivo* substrate BamA. We note that while this study was under review, similar work was presented in a preprint report revealing that residue H246 is important for BepA function (44) and that the active-site plug can be tethered using the identical residues reported here, leading to switchable proteolytic activity on the LptD substrate and the *in vitro* substrate α-casein. This confirms our results and suggests that the plug may act in an autoregulatory fashion and must relocate to facilitate access for the substrate and subsequent proteolytic activity.

We also identified specific residues in the pocket and cavity formed by the TPR that are important for function. The TPR cavity was previously shown to be the site of interaction with the Bam complex (30), and here, we demonstrate that a specific conserved arginine residue within the cavity is important for function and for BepA-mediated degradation of BamA under conditions of stress. Based on previous evidence (30) and the lack of BamA degradation by the R466A derivative, we suggest that the TPR cavity is likely to be the interaction site for docking with the Bam complex; however, this will require further study. While the importance of R280 and R466 for BepA function is clear, the role of the conserved residues D347 and R470 is less so. The D347R and R470A derivatives of BepA were detected at lower levels in whole-cell lysates than the WT protein, which may explain why these proteins were less able to fully complement the CPRG permeability defect of *bepA* cells. The decreased levels of these derivatives could be due to increased instability or increased autoproteolysis, but this result suggests that they may be important for the function of the TPR pocket and cavity.

**FIG 7:**
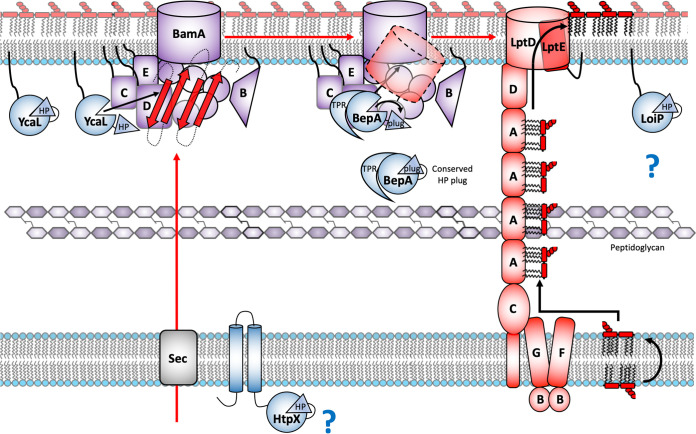
Regulation of the stages of membrane protein biogenesis by the E. coli HP plug M48 metalloproteases. Model for the proteolytic quality control of different stages in integral membrane protein biogenesis by the four E. coli M48 metalloproteases, HtpX, YcaL, BepA, and LoiP, each of which contains the conserved regulatory HP active-site plug. HtpX is an IM-localized M48 metalloprotease targeting misfolded integral membrane proteins; however, the targets remain elusive. YcaL is an OM-localized lipoprotein specifically targeting Bam-associated, unfolded OMPs, whereas BepA is a periplasmic metalloprotease targeting the next stage in OMP biogenesis, Bam-engaged partially folded β-barrels (22). Lastly, LoiP is another OM-localized lipoprotein; however, LoiP substrates remain uncharacterized. All four E. coli M48 metalloproteases encode a conserved regulatory active-site plug mechanism and appear to be involved in proteolytic quality control of specific stages in integral membrane protein biogenesis. (Based on and updated from reference 22.)

M48 metalloproteases are found in all domains of life. In humans, loss of function is associated with several diseases, such as Hutchinson-Gilford progeria, mandibuloacral dysplasia, and generalized lipodystrophy (45). In *Drosophila*, the enzymes are associated with spermatid maturation and a germ cell migration (46, 47). In Saccharomyces cerevisiae, they are required for the maturation of the α-factor mating pheromone (48). Our HMM analyses reveal that all of these proteins contain the conserved active-site plug residues, and therefore, we anticipate that these proteins may be autoregulated by a common H-P-motif active-site plug mechanism, similar to that of BepA, which is displaced upon substrate binding to induce an active state. These residues are indeed also conserved in the E. coli M48 metalloproteases, and we therefore expect that YcaL, LoiP, and HtpX are likely regulated by an active-site plug (Fig. 7). However, these observations do not shed light on the substrate recognition mechanisms of these proteins. For example, while the TPR domain of BepA is required for Bam complex interaction and substrate recognition, the three remaining metalloproteases of E. coli lack the TPR (22, 49), even though YcaL has also been shown to target BAM-engaged substrates that have yet to fold (22). Considering that it lacks the TPR domain (30), YcaL must recognize the complex and the stalled substrate through a different mechanism, revealing an interesting area of future study. We expect that our characterization of the BepA active-site plug regulatory mechanism will be important for the understanding and further study of the M48 metalloproteases in a wide range of other organisms.

## MATERIALS AND METHODS

### Expression and purification of BepA.

The BepA open reading frame, including N-terminal signal peptide, was codon optimized for expression in E. coli and cloned into the IPTG (isopropyl-β-d-thiogalactopyranoside)-inducible vector pET20b fused to a C-terminal His_6_ tag (a service provided by GenScript). This vector was transformed into E. coli DE3 cells and used for recombinant-protein production. Briefly, overnight cultures grown in LB medium at 37°C were used as the inoculum for autoinduction medium supplemented with 10 μM ZnCl_2_. The resulting cultures were grown at 37°C to an optical density at 600 nm (OD_600_) of ∼0.8 before the temperature was changed to 18°C for ∼18 h. Cells were harvested by centrifugation, and cell pellets were stored at −80°C.

To purify His-tagged BepA, cell pellets were resuspended in buffer A (20 mM imidazole, pH 7.5; 400 mM NaCl) supplemented with 0.05% Tween 20 and lysed by sonication. Cell lysates were clarified by ultracentrifugation and then incubated with Super Ni-NTA (nickel-nitrilotriacetic acid) agarose resin (Generon) at 4°C with gentle agitation overnight. The incubation mixture was centrifuged briefly, the supernatant was removed, and the resin was resuspended in buffer A before being loaded onto a gravity flow purification column. The resin was washed extensively with buffer A and then with 20 ml of buffer A supplemented with 50 mM imidazole before being washed with buffer B (400 mM imidazole, pH 7.5; 400 mM NaCl; 2% glycerol). BepA protein, eluted in buffer B, was dialyzed against buffer C (20 mM MES [morpholineethanesulfonic acid], pH 6.5; 5 mM EDTA) at 18°C for 6 h (to remove metals copurified with BepA protein) and then dialyzed extensively with sequential buffer changes against buffer D (like buffer C but lacking EDTA and instead supplemented with 10 μM ZnCl_2_ and 150 mM LiSO_4_) at 18°C. BepA protein was concentrated to ∼60 mg/ml by ultrafiltration and then further purified on a HiLoad Superdex 200 26/600 column (GE Healthcare) equilibrated in buffer D. Fractions containing pure BepA protein were pooled and concentrated to 35 mg/ml for use in crystallization trials.

### Crystallization and determination of BepA structure.

Purified recombinant BepA was used with proprietary crystal screens (supplied by Molecular Dimensions and Jena Bioscience) in sitting-drop crystallization experiments using 2 μl of protein solution and 2 μl of crystallization mother liquor at 18°C. Large crystals were obtained in 0.1 M Na HEPES, pH 7.0, and 8% (wt/vol) polyethylene glycol (PEG) 8000 and grew within 30 days. Crystals were cryoprotected by stepwise addition of mother liquor supplemented with 25% ethylene glycol prior to flash freezing in liquid nitrogen.

Protein crystals were used in X-ray diffraction experiments at the Diamond Light Source synchrotron facility (Oxford, United Kingdom). Data for single anomalous diffraction (SAD) experimental phasing were collected at a wavelength of 1.28 Å and were processed using XDS. A single atom of Zn^2+^ (copurified with BepA) was identified using SHELXD. This initial map was used for autobuilding with Phenix. Models were improved by iterations of refinement using Phenix and manual manipulations in COOT.

### Conservation analysis.

The consurf server was used to analyze conservation of surface residues. A multiple alignment of BepA homologues was generated using Clustal Omega and submitted to the consurf server along with the BepA pdb file as a basis for conservation analysis. In addition, the amino acid sequences of the four M48 metalloproteases encoded by E. coli were used to generate a multiple alignment by using Clustal Omega and visualized using ESPript 3.0 (http://espript.ibcp.fr) (see Fig. S4 in the supplemental material). Last, Pfam was used to visualize conservation within the M48 metalloprotease family through use of the HMM logo and the Skylign web server (http://skylign.org) (50–52).

### Mutagenesis of *bepA*.

Mutations in *bepA* were created using a PCR-based site directed mutagenesis approach using the pET20b::*bepA*::His_6_ vector as the template. Briefly, pET20b::*bepA*::His_6_ was used in 18 cycles of PCR using the Phusion polymerase (NEB) as described by the manufacturer but using complementary primers containing the desired mutation flanked by at least 15 bp of sequence (Table S1). As a negative control, replica reactions were set up and the polymerase omitted. Template DNA was then digested by addition of 20 U (1 μl) DpnI restriction enzyme (R0176S; NEB) and incubation at 37°C for 1 h. The reaction mixture was then used to directly transform NEB DH5α high-efficiency competent cells. Mutations were confirmed by plasmid isolation and Sanger sequencing (Source Bioscience).

### Functional screening of mutant *bepA*.

Parent or Δ*bepA* cells were first transformed with the appropriate pET20b::*bepA*::His_6_ vector and stored as glycerol stocks at −80°C. Starter cultures were generated by growth overnight (∼16 h) at 37°C with aeration in LB broth (10 g/liter tryptone, 5 g/liter yeast extract, 5 g/liter NaCl) supplemented with 100 μg/ml carbenicillin. Cells were normalized to an OD_600_ of 1 and then 10-fold serially diluted before 1.5 μl was spotted onto the relevant LB agar plates. Cells were then incubated at 37°C overnight (∼16 h), and the plates were photographed for the record. Cells were screened on LB agar plates supplemented with 100 μg/ml carbenicillin and vancomycin at the stated concentrations and 2 mM TCEP [tris(2-carboxyethyl)phosphine] where stated.

### Western immunoblotting.

To examine the expression of BepA in Δ*bepA* or parent E. coli, cells were grown as described for the experiments for functional screening of mutant BepA. For analysis of BepA-mediated degradation of BamA, cells were grown for 16 h at 37°C in M9 minimal medium supplemented with 0.1% Casamino Acids, 0.4% glucose, and 2 mM MgSO_4_ but with CaCl_2_ omitted and 2 mM TCEP added where indicated. The OD_600_ of the cultures was recorded, and cells were isolated by centrifugation and then resuspended in Laemmli buffer so that the numbers of cells in all samples were equivalent. Samples were boiled for 10 min, followed by a brief centrifugation step, before being resolved by SDS-PAGE and subjected to Western blotting using anti-His_6_ antibodies (631212; TaKaRa) or anti-BamA POTRA antiserum (32) as the primary antibody and horseradish peroxidase (HRP)-conjugated anti-rabbit immunoglobulin (Sigma-Aldrich: A6154) antibodies as secondary antibodies for detection with the ECL system. Samples were loaded in duplicate and subjected to SDS-PAGE simultaneously, followed by Coomassie staining and visualization.

### CPRG permeability assay.

Following double transformation with the relevant pET20b::*bepA*::His_6_ plasmid and the pRW50/CC-61.5 *lac* reporter plasmid (36), cells were grown to mid-exponential phase (OD_600_, 0.4 to 0.6) in LB broth with aeration and harvested by centrifugation. Cells were resuspended in LB broth to an OD_600_ of 0.1, and 5 μl was used to inoculate 96-well culture plates containing 150 μl LB agar supplemented with CPRG (chlorophenol red-β-d-galactopyranoside; Sigma) (20 μg/ml), carbenicillin (100 μg/ml), and tetracycline (15 μg/ml). The 96-well plates were incubated at 30°C, and the optical density at 300 to 800 nm was monitored every 20 min for 48 h. By using the absorbance of LacZ^−^ strains unable to turn over CPRG, we created an estimating function that predicts the expected absorbance due to cell growth at 575 nm (CPR peak absorbance) using the absorbance at 450 nm and 650 nm. This model accurately predicts the growth-related absorbance at 575 nm with residuals ranging typically within ±0.02. By subtracting the actual absorbance at 575 nm, from the expected growth-related absorbance we derive the CPRG turnover score, which is exclusive to cell membrane permeability. For both expected and measured absorbance at 575 nm, the time point of 24 h postinoculation was used.

### LPS labeling, lipid A isolation, and analysis.

Labeling of LPS, lipid A purification, thin-layer chromatography (TLC) analysis and quantification were done exactly as described previously (41). Briefly, starter cultures were incubated at 37°C overnight with aeration in LB broth supplemented with 100 μg/ml carbenicillin. Starter cultures were then subcultured in 5 ml LB broth supplemented with 100 μg/ml carbenicillin, and the experiment was completed precisely as described previously, including the addition of the positive control, in which the parent strain was exposed to 25 mM EDTA for 10 min prior to harvest of cells by centrifugation in order to induce PagP-mediated palmitoylation of lipid A (41). Experiments were completed in triplicate, and the data generated were analyzed as described previously (41).

### Data availability.

The BepA X-ray structure has been deposited in the PDB with the accession number 6SAR.

## Supplementary Material

Supplemental file 1
