# Supplementary material for: Structure-Function Characterization of the Conserved Regulatory Mechanism of the Escherichia coli M48 Metalloprotease BepA
Source: J Bacteriol. 2020 Dec 18;203(2):e00434-20. doi: 10.1128/JB.00434-20 (PMC7950410; doi:10.1128/JB.00434-20)
Supplement: Supplemental file 1 [file JB.00434-20-s0001.pdf]

Supporting information

Table S1 – Oligonucleotides used in this study

Name	Sequence	Targetted mutation
yfgC E137Q Fwd	CTGGCTTCAGTTATGGCGCACCAGATCTCCCACGTCACCCAACG	E137Q
yfgC E137Q Rev	CGTTGGGTGACGTGGGAGATCTGGTGCGCCATAACTGAAGCCAG	E137Q
yfgC H246N Fwd	CGCCGGAAATTTTATTGACTAACCCGTTGCCGGAAAGTCGTCTG	H246N
yfgC H246N Rev	CAGACGACTTTCCGGCAACGGGTTAGTCAATAAAATTTCCGGCG	H246N
yfgC R280M fwd	GATTTCTATCTGGCGAAAGCGATGACACTGGGGATGTATAATTC	R280M
yfgC R280M rev	GAATTATACATCCCCAGTGTCATCGCTTTCGCCAGATAGAAATC	R280M
yfgC R280Q fwd	GATTTCTATCTGGCGAAAGCGCAGACACTGGGGATGTATAATTC	R280Q
yfgC R280Q rev	GAATTATACATCCCCAGTGTCTGCGCTTTCGCCAGATAGAAATC	R280Q
yfgC D347R fwd	CTGGCAACGCATGGTATCTCCGTCTGGCTACTGATATCGATC	D347R
yfgC D347R rev	GATCGATATCAGTAGCCAGACGGAGATACCATGCGTTGCCAG	D347R
yfgC R466A fwd	GGCAGCCTGCAACAAGCGGCTTACGATGCGCGCATCGAC	R466A
yfgC R466A rev	GTCGATGCGCGCATCGTAAGCCGCTTGTTGCAGGCTGCC	R466A
yfgC R470A fwd	CAAGCGCGTTACGATGCGGCCATCGACCAGTTGCGCCAGC	R470A
yfgC R470A rev	GCTGGCGCAACTGGTCGATGGCCGCATCGTAACGCGCTTG	R470A
yfgC E103C fwd	GACACCGTTTCATTTTTTTCTGATCAACAACGACTGCATTAACGCCTTTGCTTTCTTTGGCGGCAACG	E103C
yfgC E103C rev	CGTTGCCGCCAAAGAAAGCAAAGGCGTTAATGCAGTCGTTGTTGATCAGAAAAAAATGAAACGGTGTC	E103C
yfgC E241C fwd	CTCGATCAGGCGCGTTACTCCTCGCGCCCGCCGTGCATTTTATTGACTCACCCGTTGCCGGAAAGTCGTCTGGCAGATG	E241C
yfgC E241C rev	CATCTGCCAGACGACTTTCCGGCAACGGGTGAGTCAATAAAATGCACGGCGGGCGCGAGGAGTAACGCGCCTGATCGAG	E241C

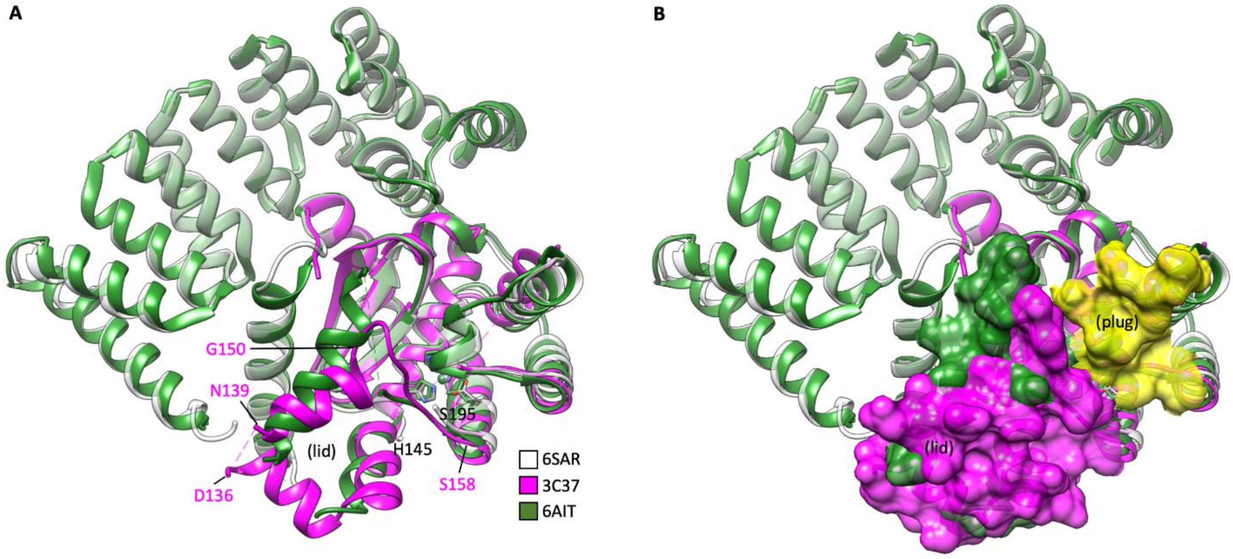

Figure S1 - Comparison of PDB: 3C37 and BepA shows the active site lid
Comparison of the *Geobacter sulfureducens* M48 protease structure (PDB: 3C37 – magenta) with that of the BepA structure presented here (PDB: 6SAR – white) and that presented previously (6AIT – green) shows the active site lid formed by the missing residues H145-S195. **A.** Alignment of 6SAR, 6AIT and 3C37 as ribbon representation **B.** Alignment of 6SAR, 6AIT and 3C37 as ribbon representation with surface representation shown for the active site plug of 6SAR (yellow) and the active site lid of 3C37 (magenta) and 6AIT (green) to demonstrate occlusion of the BepA active site.

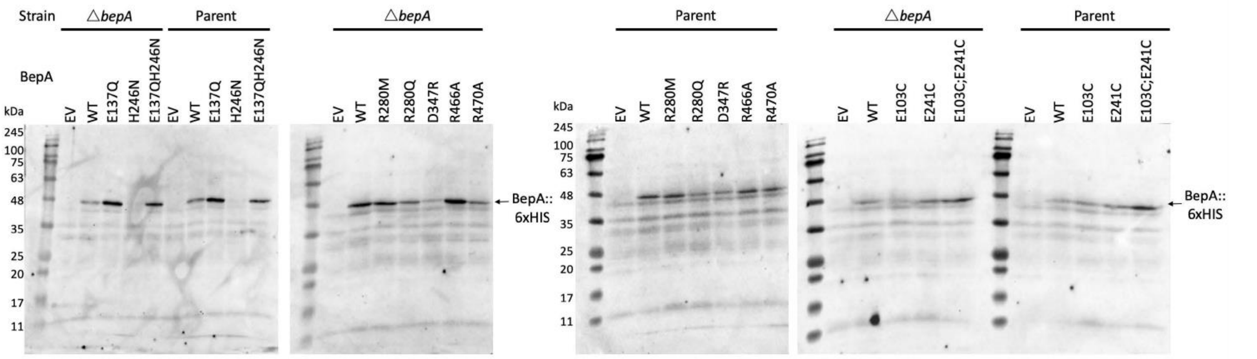

Figure S2 - Western immuno-blotting analysis of BepA::6xHis expression
Analysis of BepA expression by western immuno-blotting analysis. Cells carrying pET20b encoding WT or mutated copies of BepA in the parent or Δ*bepA* strain background were harvested and resuspended in Laemmli buffer so that the number of cells in each sample was equal. Following a brief centrifugatiuon step, proteins were separated by SDS-PAGE and transferred by western blot. The empty vector control is labelled EV. Western blotting was completed using anti-6xHis antibody raised in mice and anti-mouse::HRP secondary antibody to target the BepA::6xHis protein in samples used for vancomycin sensitivity screens.

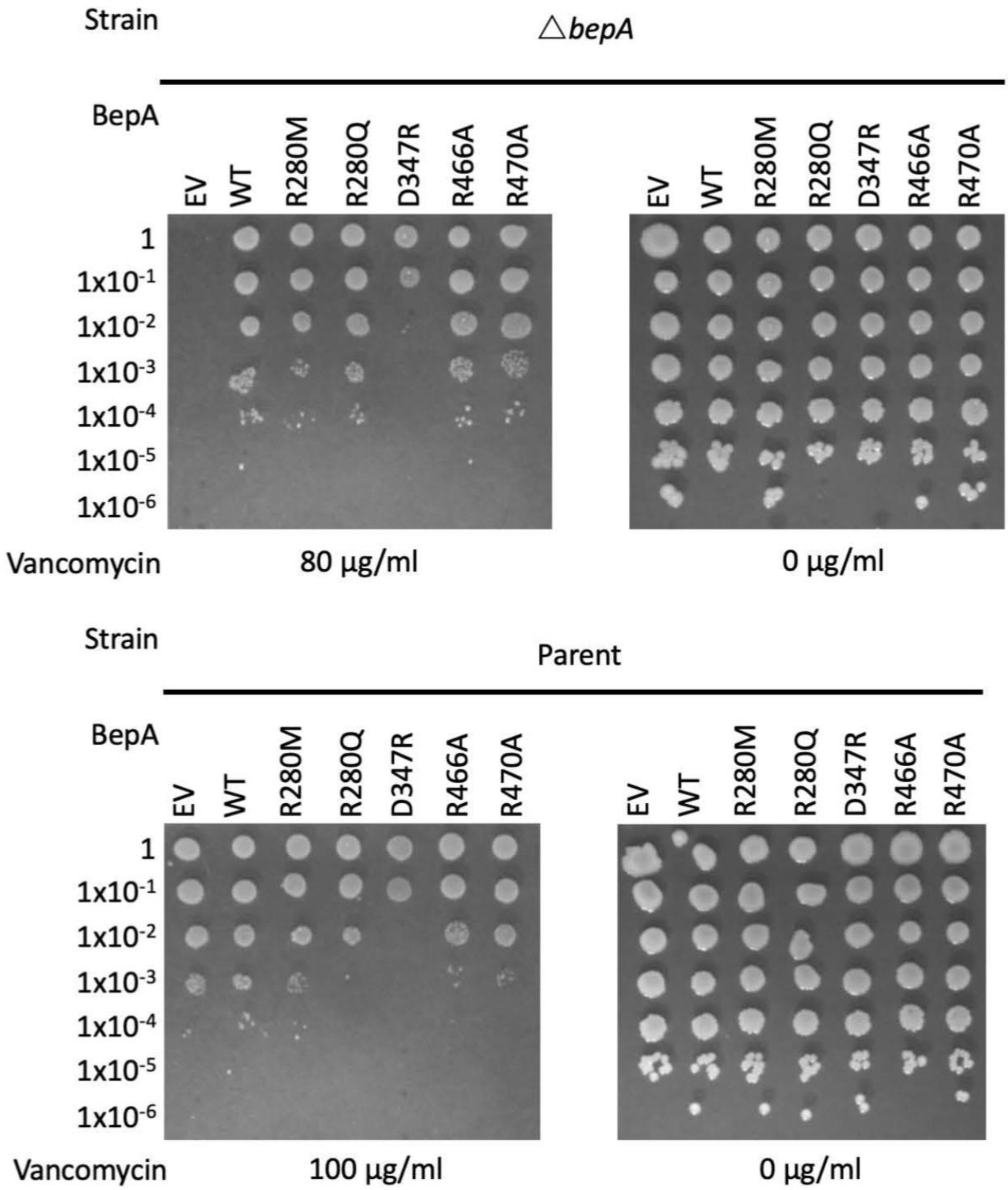

Figure S3 – Vancomycin sensitivity screen for pocket and TPR cavity mutants
Screen for vancomycin sensitivity of cells carrying pET20b encoding WT or mutant copies of BepA in the parent or Δ*bepA* strain background. The empty vector control is labelled EV. Cells are normalised to OD_{600} = 1 and ten-fold serially diluted before being spotted on the LB agar containing the indicated antibiotics (all plates contain 100 μg/ml carbenicilin additionally).
